# Epidemiology of Fascioliasis in Kermanshah Province, Western Iran

**Published:** 2018-07

**Authors:** Arezoo BOZORGOMID, Naser NAZARI, Eshrat Beigom KIA, Mehdi MOHEBALI, Homa HAJARAN, Peyman HYDARIAN, Yazdan HAMZAVI, Sara NEMATI, Mojgan ARYAEIPOUR, Mohamad Bagher ROKNI

**Affiliations:** 1. Dept. of Microbiology, Asadabad School of Medical Sciences, Asadabad, Iran; 2. Dept. of Medical Parasitology and Mycology, School of Public Health, Tehran University of Medical Sciences, Tehran, Iran; 3. Dept. of Medical Parasitology and Mycology, Faculty of Medicine, Kermanshah University of Medical Sciences, Kermanshah, Iran; 4. Dept. of Medical Parasitology and Mycology, Faculty of Medicine, Zanjan University of Medical Sciences, Zanjan, Iran

**Keywords:** *Fasciola*, Prevalence, Human, Livestock, *Lymnaea*, Iran

## Abstract

**Background:**

We aimed to determine the prevalence of fasciolosis in the definitive hosts (human and livestock) and intermediate (*Lymnaea* snails) hosts in Kermanshah Province, western Iran from 2014–2016.

**Methods:**

The study on animals was descriptive and retrospective one. All daily records of animals slaughtered in the abattoirs were analyzed. For the study of human fascioliasis, 975 serum samples were collected from different parts of Kermanshah Province and analyzed using ELISA based on excretory-secretory antigens. Moreover, 4400 *Lymnaea* snails were collected from 25 habitats. The snails were identified and examined for presence of cercariae by shedding method.

**Results:**

Fasciolosis was diagnosed in 1.7% of slaughtered animals, which was significantly greater than the other species (*P*<0.005). There was significant difference (*P*<0.001) between the prevalence of fasciolosis and seasonal pattern. As for human cases, five cases (0.5%) were positive for fascioliasis. Regarding the seropositivity to fasciolosis, no significant differences were found for age groups, sex, level of education and occupation. No *Fasciola* infection was seen in snails of the family Lymnaeidae.

**Conclusion:**

The prevalence of *Fasciola* parasite was lower compared to other provinces. This is probably due to sequential decline in rainfall and hot climate that makes conditions difficult for the snail intermediate host snails and the larval stages of fasciolid trematodes. The habitual food of people is another important point.

## Introduction

Fasciolosis is a parasitic disease caused by liver fluke species of the genus *Fasciola*, *F. hepatica*, and *F. gigantica*. This disease predominates in areas where livestock; normally sheep and cattle are raised, they being the most important reservoirs for the organism and causes economic loss due to condemnation of affected organs, fatality decreased production of meat, milk, and wool; decreased weight and infertility (1). Moreover, fasciolosis is an important zoonosis for public health with up to 2.4–17 million individuals thought to be infected worldwide and more than 180 million at risk of infection (2). Humans are accidental hosts by eating aquatic plants or drinking water contaminated with metacercaria (1).

Infected ruminants can be a significant source of human *Fasciola* infection since the livestock grazing areas is often near to the freshwater plants grown for human consumption. The prevalence of fascioliasis in various livestock has been reported 0.1% to 91.4% in Iran (3).

The species of the Lymnaeidae family are known as intermediate hosts for *F. gigantica* and *F. hepatica*. Up to now, seven species of *Lymnaea* have been identified from different parts of country include *L. stagnalis*, *L. gedrosiana*, *L. peregra*, *L. truncatula*, *L. auricularia*, *L. rufescens*, and *L. palustris*. Although, in Iran, *L. truncatula* and *L. gedrosiana* are considered as the main intermediate hosts of *F. hepatica* and *F. gigantica*, respectively, other lymnaeid species could also act as alternative intermediate hosts (4). However, in Iran, little information is available regarding the prevalence of *Fasciola* in intermediate hosts.

The first outbreak of human fascioliasis was reported in 1989 in Guilan Province (3). Since then various studies have been carried out on the prevalence of *Fasciola* infection in Iran (5–8). In addition, minor emergence of fasciolosis was observed in Kermanshah, western province of Iran in 2000 (9). In Iran, human cases of infection with both *Fasciola* species have been previously reported by morphological and molecular surveys (7, 10). In spite of this, it is difficult to distinguish *Fasciola* species under a microscope (11, 12).

At present, the prevalence of *Fasciola* infection in Kermanshah Province is still unknown. A lack of knowledge is the biggest hurdle on our understanding of the epidemiological characteristics and dynamic transmission of fasciolosis in this region. Therefore, the objective of the present study was to determine the prevalence of fasciolosis in the definitive host (human and livestock) and intermediate (*Lymnaea* snails) hosts in Kermanshah Province, west Iran.

## Materials and Methods

### Study area

**Fig. 1:**
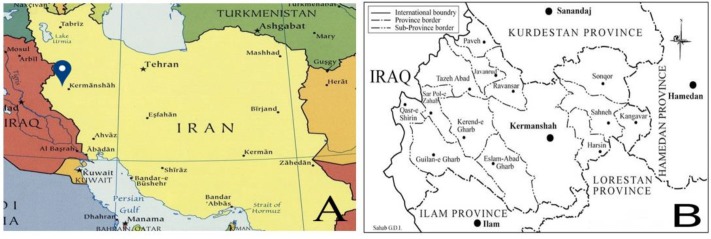
Map of Iran, showing the location of the Kermanshah Province in western country (A). Geographic location of Kermanshah Province, its cities and neighboring provinces (B)

Kermanshah Province, western Iran (lat 45.5° and 48° E, long 33.7° and 35.3° N), with an area of 24998 km^2^ is composed of fourteen districts. The annual average temperature and rainfall are 26 °C and 1935 mm, respectively. The population of the province in 2017 was about 2 million (urban: 61.7%, rural: 37.7% and the rest were non-residents) (Fig. 1). The province is located between Iranian Plateau and Mesopotamia Plain in the mountainous area and Zagross heights along with summits to cover the whole area. Parts of the slopes of a lower incline and mountainous expansions are low lands and alluvium plains. The climate of the highlands is mild in summer and cold in winter, with heavy snowfall; only the province’s western strip belongs to the warm climate.

### Ethical approval

This study was approved by the local Ethics Committee of Tehran University of Medical Sciences. The informed consent of the participants or their guardians was obtained.

### Serological examination

Overall, 975 blood samples were collected from selected subjects by randomized cluster sampling method. Following centrifugation, sera were stored at −26 °C until tested. A questionnaire was used to obtain demographic information such as age, sex, education, occupation and consumption of local freshwater vegetables. The serum samples were analyzed using ELISA method performed as previously described (7). Plates were read on by ELISA reader at an absorbance of 490 nm.

### Identification of Fasciola larval forms in Lymnaea snails

During the period from Sep 2014 to Oct 2016, 4400 *Lymnaea* snails were collected from 25 habitats (agriculture, rivers, swamps, and various streams) in the study area. Snails were first identified based on the morphological keys (4). Then each 7 to 8 snails were placed in a petri dish containing water and were placed against light for two hours or overnight in the room temperature. The snails examined for presence of cercariae by shedding method (the release of cercariae from the host snail in nature). Petri dishes were checked carefully under a dissecting microscope for the presence of *Fasciola* larvae. The cercariae were identified based on a key for identifying trematode cercariae.

### Slaughterhouse Data

We used all daily condemnation records from 178920 livestock (cattle, goats, and sheep) slaughtered in the abattoirs of different localities in Kermanshah, from Sep 2014 to Oct 2016. Fascioliasis was recognized by morphological characteristics of them. Data, such as species, gender and season, were recorded on standardized data sheets.

### Statistical analysis

Statistical analysis was done using SPSS (ver. 16, Chicago, IL, USA). Chi-square test was used for analyzing data. Cut-off was calculated as mean ± 3 SD.

## Results

During the period under study, 178920 animals (cattle 41805, sheep 125313 and goats 11802) were slaughtered in the abattoirs in Kermanshah Province. Overall, the prevalence of ruminant fasciolosis was 1.7%. Fasciolosis was significantly higher (*P*<0.05) in cattle (3%) than sheep (1.1%) and in goats (2.3%) (Table 1). There was significant difference (*P*<0.01) between the prevalence of fasciolosis and seasonal pattern. The highest prevalence rate was in the autumn (Table 2).

**Table 1:** The prevalence of fasciolosis among cattle, sheep, and goats

***Species of animal***	***Number of examined***	***Positive***	***Prevalence (%)***
Cattle	41805	1249	3.0
Sheep	125313	1438	1.1
Goats	11802	269	2.3
Total	178920	2956	1.7

**Table 2:** Seasonal distribution of *Fasciola* spp. infection in different animals

***Seasons***	***Number of examined***	***Positive***	***Prevalence (%)***
Spring	61778	719	1.2
Summer	53456	1070	2.0
Autumn	28513	672	2.4
Winter	35173	495	1.4
Total	178920	2956	1.7

**Fig. 2:**
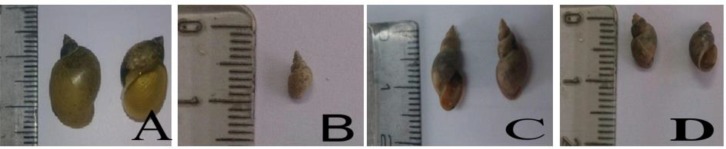
Lymnaeid snails are collected from Kermanshah Province. *L. gedrosiana* (A), *L. truncatula* (B), *L. palustris* (C), *Lymnaea* sp. (D)

Totally, 4400 lymnaeid snails were collected of which 3459 were morphologically identified as *L. gedrosiana* (78.6%), 4629 as *L. truncatula* (19.3%) and 91 (2.1%) *L. palustris*. *Fasciola* infected snails were not found at Kermanshah Province (Fig. 2).

**Fig. 3:**
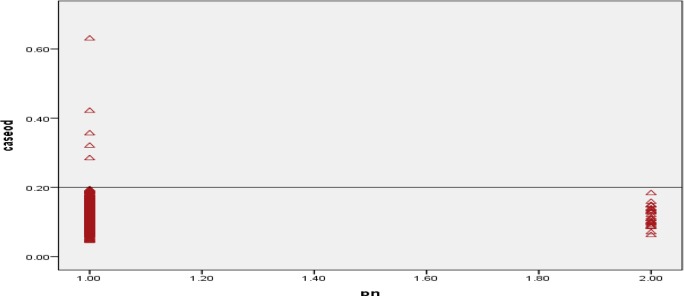
Distribution of optical density (OD) in enzyme-linked immunosorbent assay (ELISA) for *Fasciola* immunoglobulin (Ig) G of sera from individual. (1975 cases, Lanes 1), and normal controls (30, Lanes 2)

Figure 3 shows the distribution of optical density (OD) in subjects and healthy control cases. Cut-off was calculated as 0.2. Five cases (0.5%) were positive for fascioliasis by ELISA test (Fig. 3). The seroprevalence of fascioliasis among females was 0.5% and 0.36% in males. The highest positive rate was in age group of 20–29 yr old. According to job, three were housekeepers, one student, and one self-employed. The prevalence of human fasciolosis in rural and urban areas was 1.73% and 0.24% respectively. Three of seropositive cases used to eat raw vegetables.

## Discussion

Fascioliasis is a significant limiting factor on the economy of the livestock industry and human health; therefore, the development of control strategy to reduce the incidence is a priority. Now, 17 years after the first epidemic human fascioliasis was reported (9), little information is available regarding the prevalence of *Fasciola* infection in Kermanshah Province. The results of the presented study provide an overview of the occurrence of *Fasciola* spp. in this province.

In this study, the prevalence of *Fasciola* parasite in slaughtered animals was 1.7%, which were relatively lower compared with previous researches in which those in Jahrom and Lorestan were between 3.44% and 6.3%, respectively (13, 14). In old reports, the prevalence of livestock fascioliasis has been reported 0.1% to 91.4% in Iran (3, 15). Although molecular identification of *Fasciola* species was not carried out in this study, *F. hepatica* seems to be the major cause of fascioliasis in animals in this region (16).

The overall human seroprevalence as determined by ELISA was 0.5% that was lower than some studies previously conducted in some country provinces of Lorestan (1.3%) (7), Meshkin-Shahr (1.96%) (6), Isfahan (1.7%) (8), Tehran (24.8%) (5); and higher than in others such as Chaharmahal and Bakhtiyari (0.1%) (17). However, serological results may detect active or past infection because high antibody titers may remain in the human body for long period after treatment and cure.

One of the possible reasons for the decrease the prevalence infection is sequential decline in rainfall, hot climate and dried the ponds in the area in recent years that could have a significant impact on the survival of both the snail intermediate host snails and the larval stages of fasciolid trematodes (18). Moreover, effective using of anti-parasitic treatments, changes in traditional animal husbandry practices and livestock breeding methods has been reduced in all types of veterinary parasites including fasciolosis. However, the most important point is food habitual in the area where people do not like to consume water plants. Fresh-water aquatic plants such as *Nasturtium nasturtium-aquaticum* (Locally name Kuzala); *Allium ampeloprasum* are present in the area but normally people reluctant to consume them or the contamination rate of these plants is low. In addition, human infection may occur by drinking water containing viable metacercariae in areas where individuals do not have a history of freshwater plan (18).

Study on the snail intermediate hosts of fascioliasis in Kermanshah Province indicates the presence of *L. truncatula* (19.3%), *L. gedrosiana* (78.6%) and *L. palustris* (2.1%). All three species may play a role in transmission of fascioliasis (4). However, we could not detect larvae of *Fasciola* from these snails. Researchers in study on Lymnaeidae snails in Mazandaran and West Azerbaijan areas did not report any cases of *Fasciola* cercariae (19, 20). In 2001, two years after the largest disease epidemic in the province of Guilan, 1.4% of Lymnaeidae snails were infected with *Fasciola* (21). In Iran, a molecular survey in West Azerbaijan revealed that the prevalence of *Fasciola* cercariae among *L. auricularia* was 4.64% (22). A fasciolid infection rate obtained by cercarial shedding method is usually lower than those observed in nature are, because cercarial shedding is very time-consuming and may take a week or more. In addition, variable numbers of infected snails died without shedding of *Fasciola* cercariae. The utilization of molecular methods such as PCR coupled with microscopic examinations could result in a more comprehensive understanding of the susceptibility of potential intermediate hosts to *Fasciola* parasite.

## Conclusion

Although serology blood test showed very low infection rate (0.5%) but the presence of intermediate hosts and animal reservoirs might be suggested much greater infection in these areas. However, further research on the correct identification of intermediate-host species, as well as possible routes of transmission of trematode infection to humans, is needed.

## Ethical considerations

Ethical issues (Including plagiarism, informed consent, misconduct, data fabrication and/or falsification, double publication and/or submission, redundancy, etc.) have been completely observed by the authors.
